# The relationship between cumulative ecological risk and health risk behaviors among Chinese adolescents

**DOI:** 10.1186/s12889-024-17934-y

**Published:** 2024-02-26

**Authors:** Jiaojiao Wang, Yang Xie, Yi Zhang, Huiqiong Xu, Xianglin Zhang, Yuhui Wan, Fangbiao Tao

**Affiliations:** 1 Department of Maternal, Child & Adolescent Health, School of Public Health, Anhui Medical University, Hefei, Anhui China; 2 Anhui Provincial Key Laboratory of Population Health & Aristogenics, Anhui Medical University, Hefei, Anhui China; 3 Moe Key Laboratory of Population Health Across Life Cycle, Anhui Medical University, Hefei, Anhui China; 4 NHC Key Laboratory of Study On Abnormal Gametes and Reproductive Tract, Hefei, Anhui China

**Keywords:** Cumulative ecological risk, Adolescent, Health risk behavior

## Abstract

**Objectives:**

To explore the relationship between cumulative ecological risk and individual risky behavior and multiple forms of aggregated behaviors among adolescents, and examine the gender differences.

**Methods:**

A large-scale, nationally representative, and students-based investigation was conducted in rural and urban areas of eight provinces in China from October to December 2021. A total of 22 868 adolescents with an average age of 14.64 years completely standardized questionnaire in which the sociodemographic characteristics, socio-ecological risk factors and risky behaviors were used to analyze.

**Results:**

Of included students, 48.4% encountered the high level of social-ecological risk. The prevalence of breakfast intake not daily, alcohol use (AU), smoking, physical inactivity, prolonged screen time (ST) on weekdays and weekends, suicidal ideation, suicidal plan, suicidal attempt, and non-suicidal self-injury (NSSI) was 41.0%, 11.9%, 3.4%, 61.9%, 15.1%, 51.1%, 27.7%, 13.9%, 6.5% and 27.0% respectively. 22.2% of participants engaged in high-risk behaviors. All were significantly influences of increased cumulative ecological risk on individual behavior and low-risk clustering behaviors separately. The odds ratio of breakfast intake not daily, AU, smoking, physical inactivity, prolonged ST in weekday and weekend, suicidal ideation, suicidal plan, suicidal attempt, and NSSI for the adjusted model in low versus high level of cumulative ecological risk was respectively significant in both boy and girls, and the ratio of odds ratios (ROR) was separately 0.95 (*p* = 0.228), 0.67 (*p* < 0.001), 0.44 (*p* < 0.001), 0.60 (*p* < 0.001), 0.78 (*p* = 0.001), 0.83 (*p* = 0.001), 0.80 (*p* = 0.001), 0.83 (*p* = 0.022), 0.71 (*p* = 0.005), 0.75 (*p* = 0.001). Girls encountering a high level of cumulative ecological risk were more likely to engage in multiple forms of clustering risky behaviors than boys (RORs: 0.77, *p* = 0.001).

**Conclusions:**

Research and effective inventions at the social-ecological environment, based on the view of cumulative risk, are needed to promote the healthy development of behaviors in adolescence, and pay more attention to decreasing the occurrence of risky behaviours in girls than boys.

**Supplementary Information:**

The online version contains supplementary material available at 10.1186/s12889-024-17934-y.

## Introduction

Adolescents, in a heightened propensity stage for engaging in risk behaviors that can contribute to health risk behaviors (HRBs) aggregation or co-occurrence of multiple types of HRB [1, 2], are susceptible and vulnerable to reckless and risky conduct [3]. HRBs are defined as specific forms of conduct having potential threat to psychological or mental health in the present and future [4], such as substance use, insufficient physical activity, poor dietary habits, unintentional or intentional injury, non-suicidal self-injury and so on, which can increase the susceptibility to some certain diseases or ill-health [3, 5] and a series of psychological problems later [6]. Globally, disability and death in adolescence are largely attributed to risky behaviours [7]. Reportedly, 70% of premature deaths in adulthood are caused by HRBs occurring in adolescence (WHO, 2009) [8]. Decreasing the prevalence of adolescents’ risky conduct has become a priority worldwide [9].

Summarized data from the national Youth Risk Behavior Survey in America showed a marked increase in the prevalence of substance use (from 0.8% to 30.1%), the behaviors associated with an unhealthy diet (from 3.1% to 18.0%), inadequate physical activity (from 30.3% to 52.9%), long screen time (from 19.2% to 32.2), and suicide (from 0.7% to 12.5%) [10]. A longitudinal birth cohort targeting adolescents aged 15–16 years in the UK, indicated that most adolescents engaged in physical inactivity (74%), and hazardous alcohol use (34%) [11]. In early adolescence, the cumulative incidence rate of smoking and drinking each exceeded 21%, and separately more than 65% by the end of adolescence in urban South Africa [12]. Investigation data based on 1 763 high school students aged 11–19 years from Ghana found 75.4% and 18.6% of students respectively reported insufficient physical activity and substance use [13]. A cross-sectional survey among Chinese middle school students in six cities reported the prevalence of alcohol use (AU) and excessive screen time (ST) were both over 16%, nearly a third of students involved in non-suicidal self-injury (NSSI), and 15% of students had suicidal behavior (SB) [14]. These survey data suggest that HRBs in adolescence are at a highly prevalent level and have become a major public concern in the health field worldwide.

Multiple risk behaviors commonly co-occur simultaneously with the way of clustering [15]. In early literature on behavior clustering among adolescents, for example, MacArthur, et al., 30% of adolescents participated in three to five risky behaviors simultaneously, and 6.2% of adolescents with AU reported seven or more forms of HRB [11]. Most of the students in Vietnam from cross-sectional research had a cluster of at least two HRBs and approximately half with three HRBs [16]. Survey data from our research group, included 22628 middle students in China whose multiple behaviors were divided into four categories based on the latent class model [14].

Additionally, the occurrence of risk behaviors, characterized by covariation among adolescents, can lead to a “risk behavior syndrome” which indicated engaging in one problem conduct increases the occurrence of other forms of problem behavior [17]. For example, marijuana use obviously increased the behaviors related to suicide (suicidal ideation, suicidal plan, and repeated attempted suicide) [18]. Adolescents with alcohol use had a higher risk of substance abuse and sexual risk behavior [11]. Given the clustered and synergistic nature of adolescent risk behavior, clustering models that focus on multiple behaviors to classify them may play an effective role in promoting healthy adolescent development. The covariation trend of adolescent risk behavior, is ascribed to the shared social-ecological environment in which socially organized opportunities and normative expectations could be offered to learn and involve in different forms of risk behaviors [19].

Different scholars, from different perspectives, have proposed different theoretical models of risky behaviors in adolescents. For example, the problem behavior theory indicated the interaction of the social environment system, individual characteristics system, and behavior system, which jointly act on adolescents’ behavioral development and lead to unhealthy outcomes [17]. In the theory of planned behavior (TPB), attitude, subjective norm, and perceived behavioral control were thought to be determinant factors of intent that were regarded as a major determinant of behavior [20]. Viewed from double jeopardy, multiple socioeconomic stressors experienced by adolescents may be harmful [21, 22], and resulted in the occurrence of problem behaviors [23, 24]. The theory of compensatory cognition, based on compensatory health faith, pointed out that certain unhealthy behaviors could be compensated by engaging in other behaviors [25]. Based on the perspective of social ecology, an individual was nested in a series of interacting environment systems (individual, family, school, community, policy, culture, time) and whose conduct was constrained by multiple factors which affect jointly the development of adolescents [26]. Risk factors from social ecology show a strong co-occurrence. Exploring risk factors only from a single dimension could not reveal the real environment which may bring about excessive inference [27]. Additionally, an individual tends to be more sensitive to more risk factors which contributes a greatly negative impact on the development and adaptability in adolescence [28]. Therefore, on the basis of the ecological systems theory, to examine the risk factors for adolescent-initiated HRBs within micro- and meso- (natural environment support from individual, family, and school, and interpersonal environment support from school), exo- (community environment and safety), macro- (policy and culture), and chronosystem (change in family structure), is probably more in line with adolescents in the true adversity. In addition, there is a need to explore the social-ecological risks from multiple dimensions and HRBs.

Previous researches, mainly concentrated on a single or few dimensions of risk factors to explore the association between social ecological risk and behavior [29–31]. The influences of risk and protective factors at different levels (e.g., family, school, company, community) on the multiple behavioral problems have been confirmed [32, 33]. Concerning the co-occurrence of risk factors, based on previous literature, the current study started from a cumulative risk perspective to capture exposure to risk factors and reveal real adversity the in adolescents. We aimed to assess the association between cumulative social-ecological risk from seven dimensions and multiple forms of risky behavior. In addition, the present study measures the latent class clustering of HRBs based on the properties of the aggregate of multiple forms of HRBs. We further examine the relationship between cumulative social-ecological risk and latent category clustering of HRBs, and gender difference.

## Methods

### Participants and study design

From October to December 2021, based on multistage cluster sampling, we conducted a sample investigation at eight first-tier and second-tier cities (Shenzhen, Chongqing, Zhengzhou, Shenyang, Kunming, Xuzhou, Taiyuan and Nanchang) in China that had good cooperation intention with our research team. In each selected city, two schools including one junior and senior high school apart from village and city were taken into consideration. In each school, more than 200 students from each grade were chosen. A total of 22 868 adolescents aged 14.64 were recruited to carry out questionnaire survey.

The data collection and design in present study were approved by the Ethics Committee of Anhui Medical University (Ethical NO.20200965). All participants and their parents or guardian signed the informed consent for inclusion before the survey administration.

### Measurement of sociodemographic characteristics, social-ecological risk factors and HRBs

#### Social-demographic characteristics

Demographic variables containing age, grade, gender (boys/girls), registered residence (rural/urban), paternal/maternal education level (primary school degree or less, junior high or senior high school or more) and self-reported family economy (worse, general or better), regional economic level (first-tier/second-tier cities).

#### Social-ecological risk factors

The exposure of social-ecological risk was assessed with the simplified rating questionnaire which simplified by the Social Ecological Risk Assessment Questionnaire of adolescents involving seven dimensions (individual, family, school, community, culture, policy and time dimension) [34]. The risk factors from individual dimension, were respectively measured by 3-item, e.g., “You are confident to effectively deal with any emergency”. Family risk factors in the past six-month were assessed with 3 items, e.g., “Your parent often blame you owing to something”. The exposure evaluation of school and community risk were separate performed by 4 items, e.g., “You can fit in school life well”, “You live near the road including where the noise disturbs your rest”. Policy factors were measured by 5-item, e.g., “Penalties are laid down used to administrate the behavior of damaging the public property in your school”. The questionnaire comprised 3-item respectively related to time and culture, e.g., “Your parents divorced in the past three years”, “You would buy anything expensive as long as you like.” The answer to each item consisted of five choices, 1 = “completely consistent”, 2 = “basically consistent”, 3 = “somewhat consistent”, 4 = “basically inconsistent”, 5 = “completely inconsistent”. Score of each item was summed to create a total score. The total score, ranged from 25 to 75, had a good internal consistency with a significant Cronbach’s α coefficient of 0.79 in the present research, and was dichotomized at the 50th percentile to evaluate the exposure level of social-ecological risk. As a sensitivity analysis, the social-ecological risk was also dichotomized at the 33th percentile to determine variation in the results as a consequence of binary decision.

#### Health risk behavior

Ten types of HRBs including breakfast intaking not daily, AU, smoking, insufficient physical exercise, prolonged ST in weekday or weekend, suicidal ideation, suicidal plan, suicidal attempt, and NSSI were estimated. Breakfast intaking was evaluated by asking “During the past a week, how many days did you eat breakfast?” with eight response options from 1 = 0 day to 8 = 7 days. For the item, choosing 8 was coded as no (everyday) and other options were coded as yes (not daily). AU and smoking were assessed respectively by asking “During the past a moth, how many days did you drink at least one glass of wine?”, and “During the past a moth, how many days did you smoke cigarettes?” with response options of 1 = 0 day, 2 = 1-2 days,3 = 3–5 days, 4 = 6-9 days, 5 = 10 = 19 days, 6 = 20–29 days and 7 = 30 days”, choosing 1 was separate coded as no and other option were separately coded as yes. The validity of adolescent self-reported data related to breakfast intaking, AU and smoking has been evaluated [19, 35]. Inadequate physical exercise, was assessed by asking “During the past a month, how many days did you engage in exercise, such as sit-ups, or weigh-lifting?” with the responses ranging from 0 to 7. Inadequate physical exercise was defined as less than three-day per week, with assessed validity [36]. ST on weekday or weekend was respectively measured by asking “During the past 30 days, how much time did you spend watching video, computer games or engaging in something not related to studying with electronics (such as TV, smart phone, iPad).” with responses from 1 = little to 6 = more than four hours. Two hours or more were defined as prolonged ST in weekday or weekend [37]. Suicidal ideation, suicidal plan and suicidal attempt was separately defined as yes in response to three questions “During the past a year, had you ever considered killing yourself?”, “During the past a year, had you ever made plans of suicide?”, “During the past a year, had you ever engaged in suicidal behavior?” NSSI, assessed with 12-item [38], e.g., “During the past 12 months, have you ever intentionally bitten yourself?”, was dichotomized (frequency of any NSSI of 0 versus at least 1 as no or yes). In the current study, the internal consistency of NSSI and all ten risk behaviors was respectively 0.83 and 0.77.

### Statistical analysis

All statistical analyses in the present study were conducted with SPSS version 23.0 (SPSS, Chicago, IL, USA) and Mplus version 7.0. Sociodemographic data, social-ecological risk factors and HRBs were separately describe in the total sample, girls and boys. Gender differences were compared with Chi-square test for categorical variables and ANOVA analyze for continuous variables. The categories of clustered behaviors were identified with the latent class analysis. Logistic regression analysis was used to examine the relationships between the level of social-ecological risk and different HRBs or clustered HRBs categories. In the adjustment model, we adjusted for age, grade, registered residence, parents’ educational level, self-reported family economy to examine the correlations of social ecological risk and HRBs. Furthermore, we also dichotomized the total score of social ecological risk at 33th percentile as a sensitivity analysis.

Gender differences in the relationships were examined with ratio of two odds ratios (ROR). All *p*-values were two-side, and *p*-value < 0.05 indicated significant statistical significance.

## Results

### Descriptive statistics of sample

Of the 22 868 students, the mean age was 14.64 (SD = 1.77), and 50.6% were boys. Classified by 50 percent of cumulative ecological risk scores, 51.6% and 48.4% of participants were respectively in low and high-risk level. The significant differences were shown on registered residence, self-reported family economy, registered region between different genders. there was no significant difference in the distribution of other variables between genders. There were specific details and sociodemographic characteristics about total sample and gender differences in Table 1.

**Table 1:** Descriptive statistics of participants and group comparisons, data shown as n (%)

Variables	Total (*n* = 22 868)	Boys (*n* = 11 578)	Girls (*n* = 11 290)	*p*-value
Age (Mean ± SD)	14.64 ± 1.77	14.66 ± 1.78	14.63 ± 1.75	0.343^a^
Grade				0.495^b^
Middle school	11 840(51.8)	6 061(52.3)	5 779(51.2)	
High school	11 028(48.2)	5 517(47.7)	5 511(48.8)	
Registered residence				< 0.001^b^
Rural	7 981(34.9)	4 019(34.7)	3 962(35.1)	
Urban	14 887(65.1)	7 559(65.3)	7 328(64.9)	
Paternal education				0.046^b^
Primary school degree or less	2 890(12.6)	1 446(12.5)	1 444(12.8)	
Junior high school	8 733(38.2)	4 466(38.6)	4 267(37.8)	
Senior high school or more	11 245(49.2)	5 666(48.9)	5 579(49.4)	
Maternal education				0.210^b^
Primary school degree or less	4 331(19.0)	2 185(18.9)	2 146(19.0)	
Junior high school	8 350(36.5)	4 296(37.1)	4 054(35.9)	
Senior high school or more	10 187(44.5)	5 097(44.0)	5 090(45.1)	
Self-reported family economy				< 0.001^b^
Better	3 441(15.0)	1 907(16.5)	1 534(13.6)	
General	16 656(72.8)	8 054(69.6)	8 602(76.2)	
Worse	2 771(12.1)	1 617(13.9)	1 154(10.2)	
Level of cumulative ecological risk score				0.175^b^
Low	11 789(51.6)	6 020(52.0)	5 769 (51.1)	
High	11 079(48.4)	5 558(48.0)	5 521(48.9)	
Region				< 0.001^b^
Shenzhen	3 287(14.4)	1 812(15.7)	1 475(13.1)	
Zhengzhou	2 497(10.9)	1 245(10.8)	1 252(11.1)	
Xuzhou	2 798(12.2)	1 556(13.4)	1 242(11.0)	
Nanchang	3 448(15.1)	1 698(14.7)	1 750(15.5)	
Shenyang	3 313(14.5)	1 613(13.9)	1 700(15.1)	
Taiyuan	2 670(11.7)	1 329(11.5)	1 341(11.9)	
Kunming	2 486(10.9)	1 206(10.4)	1 280(11.3)	
Chongqing	2 369(10.4)	1 119(9.7)	1 250(11.1)	

*SD* standard deviation, *HRBs* health risk behaviors

^a^ T-test

^b^ Chi-square (χ^2^)

### Association between different levels of cumulative ecological risk scores and different HRBs

According to the categories of cumulative ecological risk scores on different HRBs, the number and proportion of participants were shown in Table 2. Compared with the low cumulative ecological risk group, participants in the group of high cumulative ecological risk were significantly related with eating breakfast not daily, AU, smoking, inadequate physical exercise, prolonged ST in weekday and weekend, with OR (95%*CI*) of 2.05 (1.94–2.16), 2.41 (2.21–2.62), 2.86 (2.44–3.35), 1.22 (1.16–1.29), 2.02 (1.87–2.17), and 2.16 (2.05–2.27) (Model 1 in Table 2). There were also significant trends toward increased suicidal ideation, suicidal plan, suicidal attempt, and NSSI with different categories of cumulative ecological risk scores (Model 1 in Table 2). After adjusting for sociodemographic factors (age, gender, grade, registered residence, educational levels of parents, Self-reported family economy and economic level), the similar associations were still found between cumulative ecological risk and HRBs (Model 2 in Table 2).

**Table 2:** Number, proportion and odds ratio of different HRBs by different cumulative ecological risk levels in total samples

Variables	N (%)		Model 1			Model 2		
	low cumulative ecological risk	high cumulative ecological risk	OR	95%*CI*	*p*-value	OR	95%*CI*	*p* -value
Breakfast								
7 days	7 941(67.4)	5 560(50.2)	1.00			1.00		
Eat, not daily	3 848(32.6)	5 519(49.8)	2.05	1.94–2.16	< 0.001	2.01	1.90–2.13	< 0.001
AU								
No	10 895(92.4)	9 250(83.5)	1.00			1.00		
Yes	894(7.6)	1 829(16.5)	2.41	2.21–2.62	< 0.001	2.30	2.11–2.51	< 0.001
Smoking								
No	11 571(98.2)	10 531(94.9)	1.00			1.00		
Yes	218(1.8)	566(5.1)	2.86	2.44–3.35	< 0.001	2.56	2.17–3.02	< 0.001
Physical exercise								
Sufficient	4 763(40.4)	3 950(35.7)	1.00			1.00		
Insufficient	7 026(59.6)	7 129(64.3)	1.22	1.16–1.29	< 0.001	1.20	1.13–1.27	< 0.001
Prolonged ST in weekday								
No	10 511(89.2)	8 898(80.3)	1.00			1.00		
Yes	1 278(10.8)	2 181(19.7)	2.02	1.87–2.17	< 0.001	2.08	1.92–2.24	< 0.001
Prolonged ST in weekend								
No	6 844(58.1)	4 330(39.1)	1.00			1.00		
Yes	4 945(41.9)	6 749(60.9)	2.16	2.05–2.27	< 0.001	1.87	1.77–1.97	< 0.001
Suicidal ideation								
No	9 863(83.7)	6 674(60.2)	1.00			1.00		
Yes	1 926(16.3)	4 405(39.8)	3.38	3.18–3.60	< 0.001	3.71	3.47–3.96	< 0.001
Suicidal plan								
No	10 937(92.8)	8 748(79.0)	1.00			1.00		
Yes	852(7.2)	2 331(21.0)	3.42	3.15–3.72	< 0.001	3.88	3.55–4.23	< 0.001
Suicidal attempt								
No	11 427(96.9)	9 947(89.8)	1.00			1.00		
Yes	362(3.1)	1 132(10.2)	3.59	3.18–4.06	< 0.001	4.17	3.67–4.72	< 0.001
NSSI								
No	9 689 (82.2)	6 999 (63.2)	1.00			1.00		
Yes	2 100 (17.8)	4 080 (36.8)	2.69	2.53–2.86	< 0.001	2.99	2.81–3.19	< 0.001

Model 1 unadjusted model

Model 2 adjusted for age, gender, grade, registered residence, educational levels of parents, self-reported family economy, reginal economic level

*OR* odds ratio, *CI* confidence interval, *HRBs* health risk behaviors, *AU* alcohol use, *ST* screen time, *NSSI* non-suicidal self-injury

Classified by gender, Table 3 showed the above similar positive trend toward the relationship between levels of cumulative ecological risk and different HRBs in boys and girls respectively(Model a in Table 3), so as the model adjusted for sociodemographic characteristics (Model b in Table 3) that found obvious gender difference between cumulative ecological risk and a single HRB separately, with the exception of the breakfast intaking, that indicated the highest cumulative ecological risk had a stronger influence in girls than boys.

**Table 3:** Number, proportion and odds ratio of different HRBs by different cumulative ecological risk levels in different genders, and the gender ratio

Variables	Boys			Girls			Ratio of two odds ratios in boys versus girls ^b^	
	n (%)	OR (95%*CI*) ^a^	OR (95%*C*I) ^b^	n (%)	OR (95%*CI*) ^a^	OR (95%*CI*) ^b^	ROR	one-side *p*-value
Breakfast								
7 days	7 156(61.8)	1.00		6 345(56.2)	1.00			
Eat, not daily	4 422(38.2)	2.02(1.87–2.18) ^**^	1.96(1.81–2.21) ^**^	4 945(43.8)	2.08(1.93–2.24) ^**^	2.06(1.91–2.23) ^**^	0.95	0.228
AU								
No	9 777(84.4)	1.00		10 368(91.8)	1.00			
Yes	1 801(15.6)	2.28(2.06–2.54) ^**^	1.99(1.79–2.22) ^**^	922(8.2)	2.82(2.43–3.27) ^**^	2.99(2.56–3.48) ^**^	0.67	< 0.001
Smoking								
No	10 968(94.7)	1.00		11 116(98.5)	1.00			
Yes	610(5.3)	2.59(2.17–3.09) ^**^	2.16(1.80–2.59) ^**^	174(1.5)	4.73(3.22–6.96) ^**^	4.92(3.32–7.28) ^**^	0.44	< 0.001
Physical activity								
Sufficient	4 744(41.0)	1.00		3 969(35.2)	1.00			
Insufficient	6 834(59.0)	1.23(1.14–1.32) ^**^	1.19(1.11–1.29) ^**^	7 321(64.8)	1.22(1.12–1.31) ^**^	1.99(1.11–1.30) ^**^	0.60	< 0.001
Prolonged ST in weekday								
No	9 678(83.6)	1.00		9 731(86.2)	1.00			
Yes	1 900(16.4)	1.85(1.67–2.04) ^**^	1.86(1.68–2.06) ^**^	1 559(13.8)	2.26(2.02–2.53) ^**^	2.37(2.11–2.66) ^**^	0.78	0.001
Prolonged ST in weekend								
No	5 606(48.4)	1.00		5 568(49.3)				
Yes	5 972(51.6)	1.99(1.85–2.14) ^**^	1.71(1.58–1.85) ^**^	5 722(50.7)	2.35(2.18–2.53) ^**^	2.05(1.90–2.22) ^**^	0.83	0.001
Suicidal ideation								
No	9 036(78.0)	1.00		7 501(66.4)	1.00			
Yes	2 542(22.0)	3.17(2.88–3.48) ^**^	3.28(2.98–3.62) ^**^	3 789(33.6)	3.66(3.37–3.98) ^**^	4.08(3.74–4.46) ^**^	0.80	0.001
Suicidal plan								
No	10 366(89.5)	1.00		9 319(82.5)	1.00			
Yes	1 212(10.5)	3.24(2.84–3.70) ^**^	3.48(3.03–3.99) ^**^	1 971(17.5)	3.59(3.22–4.00) ^**^	4.17(3.73–4.67) ^**^	0.83	0.022
Suicidal attempt								
No	11 089(95.8)	1.00		10 285(91.1)	1.00			
Yes	489(4.2)	3.16(2.58–3.88) ^**^	3.33(2.69–4.11) ^**^	1 005(8.9)	3.86(3.32–4.49) ^**^	4.69(4.01–5.49) ^**^	0.71	0.005
NSSI								
No	8 806(76.1)	1.00		7 882(69.8)	1.00			
Yes	2 772(23.9)	2.46(2.25–2.69) ^**^	2.57(2.35–2.82) ^**^	3 408(30.2)	2.93(2.69–3.18) ^**^	3.43(3.14–3.75) ^**^	0.75	0.001

*OR* odds ratio, *CI* confidence, *ROR* ratio of two odds ratios, *HRBs* health risk behaviors, *AU* alcohol use, *ST* screen time, *NSSI* non-suicidal self-injury

^a.^ Unadjusted model

^b.^ Adjusted for age, grade, registered residence, educational levels of parents, self-reported family economy and reginal economic level

^**^ *p* < 0.001

### Latent class models of participants HRBs

There were decreasing trends toward AIC, BIC and adjusted BIC from 1 to 7-class. The AIC, BIC and adjusted BIC for 6- to 10- class were slightly better than other classes, in which a very small percentage included in a latent class of the sample couldn’t be generalized to a broader population (Table 4) [39, 40]. In 2- to 5-group, where was little difference about AIC, BIC and adjusted BIC, the entropy of 2-category model was the largest in all estimated models which was beyond the criteria of good category separation [41], that was identified as the best model to describe the distribution of HRBs in the sample.

**Table 4:** Summarized fit statistics for latent class model identification

Number of groups	AIC	BIC	Adjusted BIC	Smallest class, %	Entropy
1	237617.42	237697.79	237666.01		
**2**	**227868.45**	**228037.24**	**227970.50**	**22.0%**	**0.83**
3	225166.28	225423.48	225321.79	39%	0.62
4	223556.74	223902.35	223765.70	19%	0.61
5	222719.12	223153.15	222981.54	15.4%	0.66
6	222359.96	222882.40	222675.83	2.6%	0.67
7	222114.55	222725.40	222483.88	2.0%	0.68
8	221880.25	222579.51	222303.03	1.7%	0.69
9	221738.89	222526.57	222215.12	1.3%	0.67
10	221654.50	222530.59	222184.19	0.6%	0.67

Bolded numbers indicate the identified model

*AIC* is Akaike information criterion, *BIC* is Bayesian information criterion

**Fig. 1:**
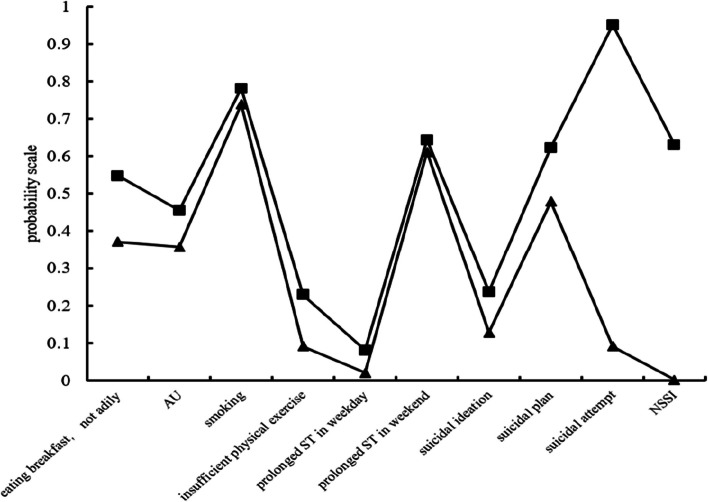
Two groups of HRBs of the best-fitting two-class pattern. AU is alcohol use; ST is screen time; NSSI is non-suicidal self-injury; ▄ low-risk behavior group, 77.8%, the prevalence of ten health risk behaviors is lower respectively; ▲ high-risk behaviors group, 22.2%, the prevalence of ten health risk behaviors is higher respectively

Figure 1 indicated the two identified latent categories for HRBs. Category 1 was recognized as a “low-risk behavior” group which contained 17 800 (77.8%) participants. In this group, 35.7% individuals reported s breakfast intaking less than 7 days in a week, 8.6% (AU), 2.2% (smoking), insufficient physical exercise (60.9%), prolong screen time in weekday (12.2%), prolonged ST in weekends (46.8%), suicidal ideation (7.9%), suicidal plan (0.1%), suicidal attempt (1.1%), NSSI (17.2%). Additionally, category 2 identified as a “high-risk” behavior group with 22.2% (5 068) adolescents of the sample was characterized by the higher percentage of participants involving in breakfast intaking less than 7-day in a week (59.3%), AU (23.6%), smoking (7.9%), inadequate physical exercise (65.2%), prolonged ST in weekday (25.4%) and weekend (66.5%), suicidal ideation (97.2%), suicidal plan (62.8%), suicidal attempt (25.6%) and NSSI (61.7%).

### Association between different levels of cumulative ecological risk and the latent class of HRBs

Table 5 showed that compared with adolescents experiencing low level of cumulative ecological risk, those whom in high-ecological risk group were more susceptible to the aggregation of high-risk behaviors (OR = 3.89, 95%*CI* = 3.63–4.17) (Model 1 in Table 5). Furthermore, the similar association occurred in the adjusted model (Adjusted for age, grade, registered residence, educational levels of parents, self-reported family economy and economic level) (Model 2 in Table 5). In addition, significant gender differences were found between social-ecological risk and the clustering of HRBs, that indicated a stronger influence in girls than boys, whether in the unadjusted model (ROR = 0.83, *p* = 0.004) or not (ROR = 0.77, *p* = 0.001).

**Table 5:** Odds ratio of different groups HRBs by different cumulative ecological risks levels in different genders, and the gender ratio

Model	Variables		Low-risk behavior class	High-risk behavior class
Model 1				
Total	OR (95%*CI*)		1.00	3.89 (3.63–4.17)
	*p*-value			< 0.01
Boys	OR (95%*CI*)		1.00	3.53 (3.18–3.92)
	*p*-value			< 0.01
Girls	OR (95%*CI*)		1.00	4.27 (3.96–4.69)
	*p*-value			< 0.01
	Ratio of two odds ratios in boys versus girls	ROR		0.83
		one-side *p*-value		0.004
Model 2				
Total	OR (95%*CI*)		1.00	4.16 (3.87–4.47)
	*p* -value			< 0.01
Boys	OR (95%*CI*)		1.00	3.58 (3.21–3.99)
	*p*-value			< 0.01
Girls	OR (95%*CI*)		1.00	4.67 (4.24–5.14)
	*p*-value			< 0.01
	Ratio of two odds ratios in boys versus girls	ROR		0.77
		one-side *p*-value		0.001

Model 1 unadjusted model

Model 2 adjusted for age, grade, registered residence, educational levels of parents, self-reported family economy and reginal economic level

*OR* odds ratio, *CI* confidence, *ROR* ratio of two odds ratios, *HRBs* health risk behaviors, *AU* alcohol use, *ST* screen time, *NSSI* non-suicidal self-injury

### Sensitive analysis

To measure the stability of the above results for the association between cumulative ecological risk and HRBs and gender differences, cumulative ecological risk scores were dichotomized at the 33 percent as an additional sensitivity analysis which were showed in the Supplementary Tables 1–3. Similar to the major consequence, the positive relationships between level of cumulative ecological risk and different HRBs were still found separate in total sample (Supplementary Table 1), girls and boys (Supplementary Table 2). The higher the cumulative risk level, the higher the occurrence risk of cluster HRBs, and girls were liable to engage in HRBs than boys with high level of cumulative ecological risk (Supplementary Table 3). Moreover, taking socio-demographic characteristics into account didn’t materially change the results for sensitive analysis (Supplementary Table 1–3).

## Discussions

In this school-based, large-scale study, the relationship between cumulative ecological risk and multiple forms of risky behavior and clustering of HRBs is investigated. The present survey revealed engaging in breakfast intaking not daily, AU, smoking, physical inactivity, prolonged ST on weekday or weekend, SB, and NSSI was each related to the level of cumulative ecological risk among the total sample, boys and girls, and a stronger association was showed in girls than boys, with the exception of breakfast intaking behavior. In addition, adolescents who encounter high levels of cumulative ecological risk are more likely to engage in cluster HRBs, and girls are more susceptible to experiencing cluster HRBs than boys.

The survey found that there was clustering of breakfast intaking not daily, AU, smoking, physical inactivity, prolonged ST on weekday or weekend, SB, and NSSI among adolescents, and two significant latent classes of HRBs were identified. The prominent disparities between two class groups were unveiled, particularly in areas such as breakfast consumption, exercise patterns, suicidal attempts, and NSSI. In the sample, the high-risk behavior group accounted for 22.2%, in which 59.3% adolescents had unhealthy breakfast consumption, 65.2% adolescents engaged in inadequate physical exercise, 25.6% adolescents had suicidal attempt and 61.7% adolescents engaged in NSSI. The incidence of various HRBs was respectively higher than the low-risk behavior group. We proposed that unhealthy breakfast consumption, insufficient physical activity, and SB may occur simultaneously, but data to elucidate this were not collected unfortunately, that need to be further explored with more research.

Influences, within the family, school, and company environment, as a part of joint efforts to account for the underlying social needs of healthy development among adolescents [42, 43], have been investigated widely in plenty of exiting studies. For example, A school-based investigation of 2 999 students in British Columbia indicated that good school connectedness reduced the prevalence of excessive drinking, and smoking [29]. Community factors, such as neighborhood (the social network, neighborhood disadvantage) [44], culture, and norms [30], greatly influenced the reckless behavior development related to adolescents. The current study extends the earlier literature by examining the impact of cumulative ecological risk from seven dimensions on multiple forms of risky behavior and clustered HRBs, and by addressing the gender differences in the association. Similar to the result of an early survey, we found a positive relationship between social-ecological risk factors clustering and multiple behaviors [45].

Significant gender differences were observed in the relationship between cumulative ecological risk and multiple behaviors. Data indicated girls were more likely to have suicidal ideation and plan than boys which was similar to the other surveys with the exception of suicidal attempts [46, 47], which might attribute to the phenomenon that girls tend to accumulate negative emotions and internalize problems due to the lack of emotional management and attention to the harmonious interpersonal relationship when faced with adverse conditions [48]. However, the stronger association between cumulative ecological risk and other externalizing behavior problems and clustering HRBs were more obvious among girls than boys. Sociocultural theory suggests that the differences in the social division of labour lead to differences in the development of sex in individuals. Traditional social divisions of labor and gender stereotypes tend to assume that males are more powerful, which requires males to undertake more challenging tasks. Compared with males, females are often thought to be more susceptible to the environment and have poorer adaption to adversity which resulted in the occurrence of risk behaviors [49]. Furthermore, the view has been verified in early empirical research, which indicated girls were more vulnerable to HRBs than boys [50]. The proportion of children intake breakfast daily is at an extremely high level, reducing somewhat in adolescence [51]. However, our study suggests that there is no gender difference in the association between cumulative ecological risk and breakfast intake, which indicating a significant correlation for both girls and boys, respectively. Further research is needed to specifically classify gender differences in the relationship between cumulative ecological risk from multiple dimensions and risky behavior.

The study sample used in the present study was favorable for a large-scale adolescents base, covering a wide area involving both rural and urban residences in eight cities in China. This survey examined the cumulative ecological risk associated with multiple forms of risky behavior and aggregated HBRs, and further examined gender differences in the relations further, based on the perspective of co-occurrence of risk factors from seven dimensions including individual, family, school, community, policy, culture and time, and multiples behaviors, respectively. However, several weaknesses in the present investigation had to be considered. First, due to the cross-sectional study design and the lack of longitudinal data, causal correlations could not be established. Second, survey data collected by self-reporting was prone to recall bias. Third, data related to unhealthy dietary diets (vegetable and fruit intake) was excluded from the study due to a large number of missing and other reasons. Fourth, the choice of the best-fitting latent class model on HRBs mainly took into account entropy, regardless of other metrics, but it may be appropriate to cluster HRBs into two latent classes, given that the large difference between the two and the number of classes.

## Conclusion

This survey extended the previous literature on the relationships between social-ecological risk factors and risky behaviors based on cumulative and covariant perspectives from seven dimensions and multiples forms of behavior, and identify the gender differences in the associations. The remarkable positive correlations were observed respectively in total sample, boys and girls. Furthermore, we revealed a stronger association in girls than boys.

### Supplementary Information

**Additional file 1:** **Supplementary 1**

## Data Availability

Owing to the appropriate protection of participants’ personal information, the original datasets used and analyzed in the current study are not publicly available, but are available from the corresponding author on reasonable request.
