# Supplementary material for: The relationship between cumulative ecological risk and health risk behaviors among Chinese adolescents
Source: BMC Public Health. 2024 Feb 26;24:603. doi: 10.1186/s12889-024-17934-y (PMC10895731; doi:10.1186/s12889-024-17934-y)
Supplement: Supplementary file 1 — Additional file 1: Supplementary 1 [file 12889_2024_17934_MOESM1_ESM.pdf]

Supplementary Table 1 Number, proportion and odds ratio of different HRBs by different cumulative ecological risk levels (classified at 33 percentile) in total samples

Variables	N (%)		Model 1			Model 2		
	Low cumulative ecological risk	High cumulative ecological risk	OR	95%*CI*	*P*-value	OR	95%*CI*	*P*-value
Breakfast								
7 days	5 667(70.1)	7 834(53.0)	1.00			1.00		
Eat, not daily	2 422(29.9)	6 945(47.0)	2.07	1.96-2.20	<0.001	2.03	1.92-2.16	<0.001
Drinking								
no	7 567(93.5)	12 578(85.1)	1.00			1.00		
yes	522(6.5)	2 201(14.9)	2.54	2.30-2.80	<0.001	2.39	2.16-2.66	<0.001
Smoking								
no	7 968(98.5)	14 116(95.5)	1.00			1.00		
yes	121(1.5)	663(4.5)	3.09	2.54-3.76	<0.001	2.73	2.23-3.33	<0.001
Physical activity								
sufficient	3 355(41.5)	5 358(36.3)	1.00			1.00		
insufficient	4 734(58.5)	9 427(63.7)	1.25	1.18~1.32	<0.001	1.22	1.15-1.29	<0.001
High ST in weekdays								
no	7 303(90.3)	12 106(81.9)	1.00			1.00		
yes	786(9.7)	2673(18.1)	2.05	1.89~2.23	<0.001	2.11	1.93-2.30	<0.001
High ST in weekends								
no	5 062(62.6)	6 112(41.4)	1.00			1.00		
yes	3 027(37.4)	8 667(58.6)	2.37	2.24~2.51	<0.001	2.04	1.93-2.16	<0.001
Suicidal ideation								
no	7 012(86.7)	9 525(64.4)	1.00			1.00		
yes	1 077(13.3)	5 254(35.6)	3.59	3.34~3.86	<0.001	3.91	3.63-4.22	<0.001
Suicidal plan								
no	7 615(94.1)	12 070(81.7)	1.00			1.00		
yes	474(5.9)	2 709(18.3)	3.61	3.26~3.99	<0.001	4.04	3.64-4.49	<0.001
Suicidal attempt								
no	7 875(97.4)	13 499(91.3)	1.00			1.00		
yes	214(2.6)	1 280(8.7)	3.49	3.01~4.04	<0.001	3.990	3.43-4.64	<0.001
NNSI								
no	6 890(85.2)	9 798(66.3)	1.00			1.00		
yes	1 199(14.8)	4 981(33.7)	2.92	2.72~3.13	<0.001	3.26	3.03-3.50	<0.001

OR, odds ratio; *CI*, confidence interval; HRBs, health risk behaviors; AU, alcohol use; ST, screen time; NSSI, non-suicidal self-injury;

Model 1 unadjusted model;

Model 2 adjusted for age, gender, grade, registered residence, educational levels of parents, self-reported family economy and reginal economic level.

Supplementary Table 2 Number, proportion and odds ratio of different HRBs by different cumulative ecological risk levels (classified at 33 percentile) in different genders, and the gender ratio

Variables	Boys			Girls			Ratio of two odds ratios in boys versus girls [b]	
	n (%)	OR (95%*CI*) [a]	OR (95%*CI*) [b]	n (%)	OR (95%*CI*) [a]	OR (95%*CI*) [b]	ROR	one-side *P*-value
Breakfast								
7 days	7 156(61.8)	1.00		6 345(56.2)	1.00			
Eat, not daily	4 422(38.2)	2.02(1.86-2.19) **	1.96(1.80-2.13) **	4 945(43.8)	2.13(1.97-2.31) **	2.11(1.94-2.30) **	0.93	0.100
Drinking								
no	9 777(84.4)	1.00		10 368(91.8)	1.00			
yes	1 801(15.6)	2.25(1.99-2.53) **	1.93(1.71-2.18) **	922(8.2)	3.51(2.91-4.25) **	3.72(3.07-4.51) **	0.52	<0.001
Smoking								
no	10 968(94.7)	1.00		11 116(98.5)	1.00			
yes	610(5.3)	2.71(2.19-3.34) **	2.21(1.78-2.75) **	174(1.5)	6.78(3.85-11.94) **	7.00(3.95-12.40) **	0.32	<0.001
Physical activity								
sufficient	4 744(41.0)	1.00		3 969(35.2)	1.00			
insufficient	6 834(59.0)	1.30(1.20-1.41) **	1.27(1.17-1.38) **	7 321(64.8)	1.19(1.09-1.29) **	1.17(1.07-1.27) **	1.09	0.074
High ST in weekdays								
no	9 678(83.6)	1.00		9 731(86.2)	1.00			
yes	1 900(16.4)	1.94(1.73-2.17) **	1.95(1.74-2.19) **	1 559(13.8)	2.22(1.95-2.53) **	2.33(2.04-2.65) **	0.84	0.026
High ST in weekends								
no	5 606(48.4)	1.00		5568(49.3)	1.00			
yes	5 972(51.6)	2.25(2.08-2.43) **	1.93(1.78-2.10) **	5 722(50.7)	2.51(2.32-2.72) **	2.16(1.99-2.35) **	0.89	0.028
Suicidal ideation								
no	9 036(78.0)	1.00		7 501(66.4))	1.00			
yes	2 542(22.0)	3.38(3.03-3.78) **	3.47(3.09-3.89) **	3 789(33.6)	3.85(3.49-4.24) **	4.28(3.87-4.73) **	0.81	<0.003
Suicidal plan								
no	10 366(89.5)	1.00		9 319(82.5)	1.00			
yes	1 212(10.5)	3.27(2.79-3.84) **	3.46(2.94-4.07) **	1 971(17.5)	3.88(3.40-4.43) **	4.49(3.91-5.14) **	0.77	0.008
Suicidal attempt								
no	11 089(95.8)	1.00		10 285(91.1)	1.00			
yes	489(4.2)	3.05(2.39-3.90) **	3.15(2.45-4.05) **	1 005(8.9)	3.76(3.12-4.52) **	4.51(3.73-5.45) **	0.70	0.013
Non-suicidal self-injury								
no	8 806(76.1)	1.00		7 882(69.8)	1.00			
yes	2 772(23.9)	2.69(2.43-2.98) **	2.82(2.53-3.13) **	3 408(30.2)	3.15(2.86-3.48) **	3.72(3.36-4.12) **	0.76	<0.001

OR, odds ratio; *CI*, confidence interval; HRBs, health risk behaviors; AU, alcohol use; ST, screen time; NSSI, non-suicidal self-injury; ** p <0.001

[a.] Unadjusted model;

[b.] Adjusted for age, gender, grade, registered residence, educational levels of parents, self-reported family economy and reginal economic level.

Supplementary Table 3 Odds ratio of different groups HRBs by different cumulative ecological risks levels (classified at 33 percentile) in different genders, and the gender ratio

Model	Variables		Low-risk behavior class	High-risk behavior class
Model 1				
Total	OR (95%*CI*)		1.00	4.33(3.98-4.71)
	P-value			<0.01
Boys	OR (95%*CI*)		1.00	3.82(3.36-4.34)
	P-value			<0.01
Girls	OR (95%*CI*)		1.00	4.83(4.32-5.42)
	P-value			<0.01
	Ratio of two odds ratios	ROR		0.79
	in boys versus girls	one-side *P*-value		0.003
Model 2				
Total	OR (95%*CI*)		1.00	4.59(4.20-5.01)
	P-value			<0.01
Boys	OR (95%*CI*)		1.00	3.83(3.36-4.37)
	P-value			<0.01
Girls	OR (95%*CI*)		1.00	5.25(4.67-5.90)
	P-value			<0.01
	Ratio of two odds ratios	ROR		0.73
	in boys versus girls	one-side *P*-value		<0.001

OR, odds ratio; *CI*, confidence; ROR, ratio of two odds ratios;

Model 1 unadjusted model;

Model 2 adjusted for age, grade, registered residence, educational levels of parents, self-reported family economy and reginal economic level.
